# Gene Expression Signatures of Porcine Bone Marrow-Derived Antigen-Presenting Cells Infected with Classical Swine Fever Virus

**DOI:** 10.3390/v17020160

**Published:** 2025-01-24

**Authors:** Liani Coronado, Miaomiao Wang, Jose Alejandro Bohórquez, Adriana Muñoz-Aguilera, Mònica Alberch, Patricia Martínez, Nicolas Ruggli, Yuliaxis Ramayo-Caldas, Llilianne Ganges

**Affiliations:** 1 Institute for Research and Technology in Food and Agriculture (IRTA), Programa de Sanitat Animal, Centre de Recerca en Sanitat Animal (CReSA), Bellaterra, 08193 Barcelona, Spain; liani.coronado@irta.cat (L.C.); wm576332031@163.com (M.W.); jabohorquezgarzon@gmail.com (J.A.B.); driana.munoz@irta.cat (A.M.-A.); monica.alberch@uab.cat (M.A.); patricia.martinez@irta.cat (P.M.); 2 Unitat Mixta d’Investigació IRTA-UAB en Sanitat Animal, Centre de Recerca en Sanitat Animal (CReSA), Bellaterra, 08193 Barcelona, Spain; 3 Classical Swine Fever World Organization for Animal Health (WOAH) Reference Laboratory for, IRTA-CReSA, 08193 Barcelona, Spain; 4 Instituto Colombiano Agropecuario (ICA), Bogotá 110911, Colombia; 5 Division of Virology, Institute of Virology and Immunology (IVI), 3147 Mittelhäusern, Switzerland; nicolas.ruggli@ivi.admin.ch; 6 Department of Infectious Diseases and Pathobiology, Vetsuisse Faculty, University of Bern, 3012 Bern, Switzerland; 7 Animal Breeding and Genetics Program, Institute for Research and Technology in Food and Agriculture (IRTA), Torre Marimon, 08140 Caldes de Montbui, Spain; yuliaxis.ramayo@irta.cat

**Keywords:** classical swine fever virus, bone marrow-derived antigen-presenting cells, transcriptomic profile, disease severity, differentially expressed genes

## Abstract

For a better understanding of classical swine fever (CSF) pathogenesis, a transcriptomic analysis was performed using porcine bone marrow (BM)-derived antigen-presenting cells (APCs) infected ex vivo with two different cDNA-derived classical swine fever virus (CSFV) strains, the low-virulence Pinar de Rio (vPdR-36U) or the lethal vPdR-H_30_K-5U. The transcriptomic profile of vPdR-36U- or vPdR-H_30_K-5U-infected versus noninfected cells revealed 946 and 2643 differentially expressed genes (DEGs), respectively. The upregulation of ISG15, CXCL-10, ADAM8, and CSF1 was found after infection with vPdR-36U, which could contribute to the generation of mild CSF forms. In contrast, cells infected with the lethal vPdR-H_30_K-5U overexpressed the immune checkpoint molecules PD-L1, CD276, and LAG3, which are involved in T-cell exhaustion and could be associated with adaptive immunity impairment. vPdR-H_30_K-5U also induced increased expression of PPBP, IL-8, IL-6, ECE1, and Rab27b, which are mediators of inflammatory responses that can be involved in cytokine storms. The TNF signaling pathway, which is related to the activation and proliferation of different subsets of immune cells, including CD4+ T cells, was notably upregulated in response to the low-pathogenicity virus. The Th17, Th1, and Th2 differentiation pathways were downregulated by the highly pathogenic virus only, supporting the role of T-cell-mediated immunity in protecting against CSFV.

## 1. Introduction

Classical swine fever (CSF) is one of the most important transboundary viral diseases of swine, affecting domestic and wild pigs and strongly impacting animal health and the pig industry. CSF is a notifiable disease according to the World Organization for Animal Health (WOAH) [1]. The disease remains endemic in Asia, as well as in various countries of Central and South America, and represents a constant risk for disease-free countries.

CSF is caused by the classical swine fever virus (CSFV), an enveloped positive-sense RNA virus that belongs to the Pestivirus genus within the Flaviviridae family. The CSFV RNA genome is approximately 12.3 kb in length and contains a single open reading frame (ORF) that encodes a polyprotein flanked by 5′ and 3′ untranslated regions (UTRs). The translated polyprotein is processed into four structural proteins (C, E^rns^, E1, and E2) and eight nonstructural proteins (N^pro^, p7, NS2, NS3, NS4A, NS4B, NS5A, and NS5B) [2]. The E^rns^ glycoprotein is an exclusive feature of pestiviruses and possesses intrinsic ribonuclease (RNase) activity that interferes with the host immune response by counteracting type I interferon (IFN) induction, with direct implications for CSF severity and pathogenesis [3,4,5].

CSF has different clinical forms that depend on the degree of virulence of the CSFV strain and on host factors, including the immune response and genetic background [1]. The clinical forms of CSF range from peracute and acute to chronic and subclinical, including persistent infections [6]. Accordingly, CSFV virulence ranges from high to moderate to low. Notably, infections with low-virulence strains may result in chronic and subclinical forms [1], which are highly relevant to this type of strain since they pose a challenge for CSFV eradication. Previously, a unique polyuridine (poly-U) sequence of 36 nucleotides in length was found in the 3′UTR of the CSFV strain Pinar del Rio (PdR) circulating in an endemic country and classified as having low virulence [7,8]. Our previous studies using reverse genetics revealed that the poly-U insertion in the 3′UTR of the PdR strain is a virulence factor in piglets and is associated with host immunity activation and a reduction in CSFV virulence [9]. In addition, in a recent study, we showed a synergistic effect of the removal of both the E^rns^ RNase function and 31 uridines in the poly-U 3′UTR insertion in the PdR strain, resulting in vPdR-H_30_K-5U. This virus generates the disproportional activation of innate immunity with high IFN-α, interleukin 12 (IL-12), and IL-8 concentrations and promotes CSFV replication in target tissues, contributing to high CSFV lethality after infection in pigs [4].

Both T-cell and humoral responses have been implicated in mediating protection against CSF disease progression and inhibiting viral replication. In addition, the antiviral role of IFN-γ against CSFV has also been demonstrated [1,10,11]. Following CSFV infection, lymphocyte apoptosis, leukopenia, platelet aggregation, bone marrow depletion affecting myelopoiesis, and megakaryocytopoiesis and thymus atrophy occur [12,13]. Notably, CSFV infection provokes an aberrant proinflammatory response (known as a “cytokine storm”), which is associated with lethal forms of the disease [4]. CSFV is known to have a specific tropism for endothelial cells and the mononuclear phagocyte system, including macrophages (Macs) and dendritic cells (DCs) [1]. Both Macs and DCs are key players in mounting innate and adaptive immune responses to CSFV [14]. Previous studies have shown high levels of CSFV replication in bone marrow hematopoietic cells (BMHCs) [6,15]. In vitro-differentiated primary cell cultures from BMHCs are highly valuable for studying DCs and Macs of the swine immune system [16,17,18,19]. Porcine BMHC cultures stimulated with recombinant porcine colony stimulating factor 1 (rpGM-CSF) are considered as a heterogeneous population of both DCs and Macs (bone marrow (BM)-derived antigen-presenting cells (APCs)) [16,18,19]. These cells have been used previously to carry out studies with different porcine viruses, such as the African swine fever virus (ASFV), porcine circovirus type 2 (PCV2), porcine reproductive and respiratory syndrome virus (PRRSV), and swine influenza virus [17,20,21,22]. High-throughput RNA sequencing (RNA-Seq) is a powerful approach for identifying and quantifying differentially expressed genes (DEGs) on a genome-wide scale. Although this approach allows the identification of gene markers that may play relevant roles in CSF pathogenesis, few studies have been conducted to decipher the host pathways and DEGs activated or suppressed during low-pathogenicity or lethal CSFV infection. In the present study, the differential gene expression signatures between low- and high-pathogenicity CSFVs were determined after infection of porcine BM-APCs. To guarantee the best interpretation of the information obtained by RNA-seq, two well-characterized viruses with similar genomic information were selected. To this end, two cDNA-derived CSFV strains obtained by modification of the PdR backbone, which induced different clinical forms of CSF (chronic or severe) in infected animals, were used [4,9].

## 2. Materials and Methods

### 2.1. Cells and Viruses

The porcine aortic endothelial cell line PEDSV.15, provided by Jörg Seebach, University of Geneva, Switzerland, was tested for the absence of pestiviruses. PEDSV.15 cells were propagated in Dulbecco’s modified Eagle medium (DMEM) supplemented with 1% sodium pyruvate, 1% nonessential amino acids, 8% horse serum, and 2% porcine serum. The vPdR-36U and the double mutant vPdR-H_30_K-5U generated and characterized in previous studies were used. While vPdR-36U has low virulence, vPdR-H_30_K-5U was found to be highly pathogenic in piglets [4,9]. Both viruses were amplified by infecting PEDSV.15 cells with a multiplicity of infection (MOI) of 0.1 50% tissue culture infectious dose (TCID_50_)/cell in the presence of 2% serum and harvested after 72 h. The virus titers were determined by endpoint dilution in PEDSV.15 cells by a peroxidase-linked assay (PLA) [23], and the TCID_50_ per milliliter was calculated by standard statistical methods [24].

### 2.2. BMHC Culture and CSFV Infection

**Figure 1:**
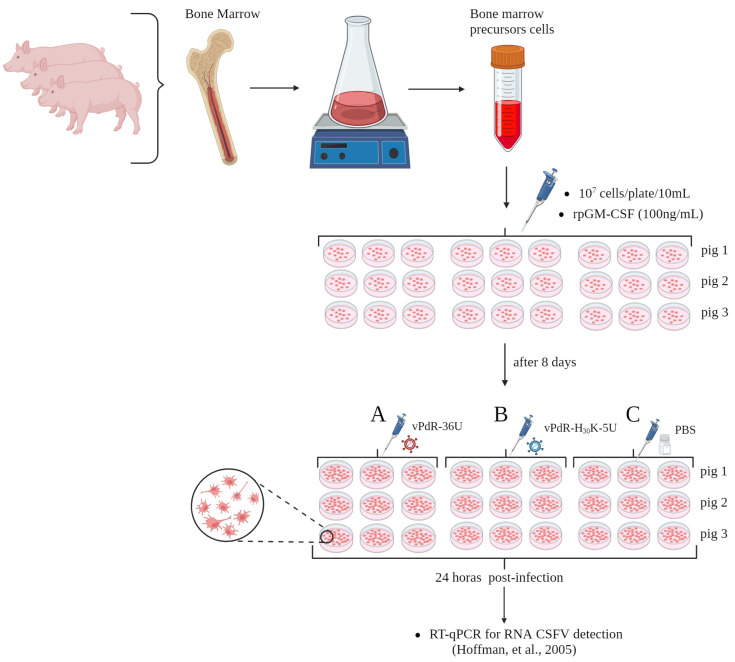
Experimental design of porcine BM-APC generation and infection with CSFV. The bone marrow hematopoietic precursor cells were obtained from the femurs of three healthy three-week-old pestivirus-free pigs. For each femur, 10^7^ cells/dish were seeded in a total of 9 dishes, and BM-APCs were generated following an eight-day protocol. Briefly, 100 ng/mL recombinant porcine GM-CSF was added to the cells three times at two-day intervals. The dishes were observed by microscopy for cell morphology and viability every two days until the end of the protocol. On day eight, the 9 dishes from each of the three femurs were split into three groups (A to C) with nine dishes per group, resulting in three sets of three dishes from each femur per group. The cells were infected with CSFV at an MOI of 0.05 TCID_50_/cell. In Group A, the cells were infected with vPdR-36U, in Group B, the cells were infected with vPdR-H_30_K-5U, and in Group C, the cells were treated with PBS. RNA was extracted 24 h postinfection, and CSFV RNA was quantified by real-time RT-qPCR followed [26] by transcriptomic analyses.

Three healthy three-week-old domestic pigs (sus scrofa) were acquired from a commercial farm in Catalonia, Spain. The animals were euthanized with 60 to 100 mg of pentobarbital per kg of body weight injected into the vena cava cranialis, according to European directive 2010/63/EU. The procedure was approved by the Ethical Committee of the Generalitat de Catalonia, Spain, in accordance with Spanish and European regulations under animal experimentation project number 12122. In addition, the methods were carried out in accordance with relevant guidelines and regulations, including the ARRIVE guidelines. BMHC precursor cells were obtained from the femurs of three domestic pigs. The cells were negative for pestiviruses, PRRSV, and PCV2. A total of 10^7^ cells per dish were seeded in a total of nine cell culture dishes for each femur. Dulbecco’s modified Eagle medium (DMEM) (Lonza, Walkesville, MD, USA) containing 8 mM l-glutamine and 200 μg/mL penicillin with 200 U/mL streptomycin and 5% FCS was incubated at 37 °C and 5% CO_2_. The number and viability of the cells were determined by staining with Trypan Blue [10]. The BM-APC culture was generated following the eight-day protocol described previously [16,19]. One hundred nanograms/milliliter (100 ng/mL) of recombinant porcine GM-CSF (R&D Systems, Barcelona, Spain) was added to the cells three times during differentiation at two-day intervals. The dishes were observed by microscopy every two days until the end of the protocol to determine the morphology and viability of the cells. On day eight, the nine dishes from each of the three femurs were distributed into 3 groups (A, B, C), resulting in three sets of dishes from the 3 different pig femurs for each group (Figure 1).

The cells were then infected with CSFV at an MOI of 0.05 TCID_50_/cell. In Group A, the cells were infected with vPdR-36U, in Group B, they were infected with vPdR-H_30_K-5U, and in Group C, the cells were treated with PBS. To determine the early transcriptional profile after CSFV infection, at 24 h postinfection, the supernatant was removed, and the cells were washed with cold PBS (Figure 1) [25]. A total of 10^7^ cells/mL were used for RNA extraction with the RNeasy Mini Kit (Qiagen GmbH, Hilden, Germany) following the manufacturer’s instructions. The RNA quantity and purity were determined with a NanoDrop ND-1000 spectrophotometer (NanoDrop Technologies, Wilmington, DE, USA), and the RNA integrity was analyzed by the Agilent Bioanalyzer-2100 (Agilent Technologies, Inc., Santa Clara, CA, USA). The presence of CSFV RNA was analyzed by real-time RT-qPCR [26]. Ct values equal to or less than 42 were considered positive. Samples in which fluorescence was undetectable were considered negative.

### 2.3. RNA-Seq

The RNA samples used for this study were of high quality (RNA integrity number (RIN) > 8). Library preparation and sequencing were performed at Microomics (IIB Sant Pau, Barcelona, Spain). For each sample, one paired-end library with an approximately 300 bp insert size was prepared with a TruSeq Stranded mRNA Kit (Illumina, Inc.; San Diego, CA, USA). Libraries were labeled by barcoding and pooled to be run on an Illumina HiSeq 3000/4000 instrument (Illumina, Inc.; San Diego, CA, USA).

### 2.4. Bioinformatic and Statistical Analyses

Quality control and basic statistics of sequence data were performed by the FastQC (v0.11.9) (https://www.bioinformatics.babraham.ac.uk/projects/fastqc/) accessed on 1 July 2024 and MultiQC v0.7 programs [27]. Low-quality reads were trimmed by Trimmomatic (v0.39) [28]. The sequencing reads were aligned to the Sscrofa11.1 pig genome reference with Bowtie2 v 2.3.5.1 (Langmead), and the gene count was determined with featureCounts v1.6.4 [29]. Differential expression analysis was performed with DESeq2 v1.24.0 [30] to compare the following three groups: (i) noninfected versus vPdR-36U-infected cells, (ii) noninfected versus vPdR-H_30_K-5U-infected cells, and (iii) vPdR-36U- versus vPdR-H_30_K-5U-infected cells. Significance was considered after adjusting the raw *p* values to control the false discovery rate (FDR) at 5% by the Benjamini–Hochberg procedure [31]. Adjusted *p* values < 0.05 were used as cutoffs to define DEGs. The most highly differentially expressed genes were selected on the basis of the 25–30 genes per group with the lowest FDR. Finally, the list of DEGs was loaded into the R package clusterProfiler v 3.12.0 to perform a gene set enrichment analysis [32] of highly overrepresented Gene Ontology and metabolic pathways. In addition, the KEEG database (https://www.kanehisa.jp) was also employed.

### 2.5. Validation of RNA-Seq Data

TaqMan probe-based RT-qPCR assays were performed to validate the DEGs identified from the transcriptome sequencing. DEGs, namely, S100A8, S100A12, CCL8, ARG1, PD-L1, and CCL2, which were upregulated in the two viruses, and CXCL10, which was upregulated in at least one of the two viruses, were selected for RT-qPCR validation. Primers and probes were designed for the selected genes with specific sequences of these genes obtained from GenBank (Table S1). The amplification was carried out with the Ag Path-ID One-step RT-PCR Kit in a total volume of 20 µL containing 0.5 μL of forward and reverse primers (10 μM), 0.5 μL of probe (20 μM), and 4 μL of RNA target (1 μg). The cycling steps for the assay included reverse transcription at 48 °C for 10 min, activation of DNA polymerase at 95 °C for 10 min, and 40 cycles of 97 °C for 2 s and 60 °C for 30 s and were performed by an Applied Biosystems^®^ 7500 Fast Real-Time PCR System. The RNA samples used for this validation were the same as those used in the RNA-seq study. The RT-qPCR assay was repeated three times, and the samples were analyzed in triplicate. The relative expression values of selected genes were calculated by the 2^−ΔΔCt^ method and normalized against the expression levels of the ß-actin gene [33].

## 3. Results

### 3.1. Similar Infection Levels with CSFV vPdR-36U or vPdR-H_30_K-5U in Porcine BM-APCs

**Figure 2:**
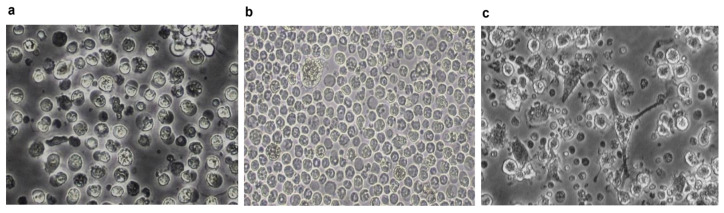
Morphology of in vitro cultured porcine BM-APCs. Microscopic analysis of BM-APCs without rpGM-CSF (**a**) and after 3 days (**b**) and 6 days (**c**) of differentiation in culture (20×).

The porcine BMHC cultures stimulated with rpGM-CSF formed clusters began to form dendrites on day three and increased in size until day eight (Figure 2). At this time, the transcriptomic profile of porcine BM-APCs revealed upregulation of the MerTK, Cadm1, XCR1, FLT3, and CSF1R genes, without any difference between vPdR-36U- and vPdR-H_30_K-5U-infected cells. In addition, transcripts encoding surface markers (CD14, CD4, CD64) were also found. CSFV infection of BM-APCs was confirmed by CSFV-specific RT-qPCR for both viruses at 24 hours postinfection. The RT-qPCR results revealed cycle threshold (Ct) values of 23.2, 23.4, and 22.9 for RNA samples 1, 2, and 3, respectively, in Group A (cells infected with vPdR-36U), whereas in Group B (vPdR-H_30_K-5U-infected cells), the Ct values were 23.6, 20.9, and 23.7 for samples 1, 2, and 3, respectively. The noninfected BM-APCs were RNA CSFV negative.

### 3.2. Gene Expression Patterns in Porcine BM-APCs After Infection with CSFV

**Figure 3:**
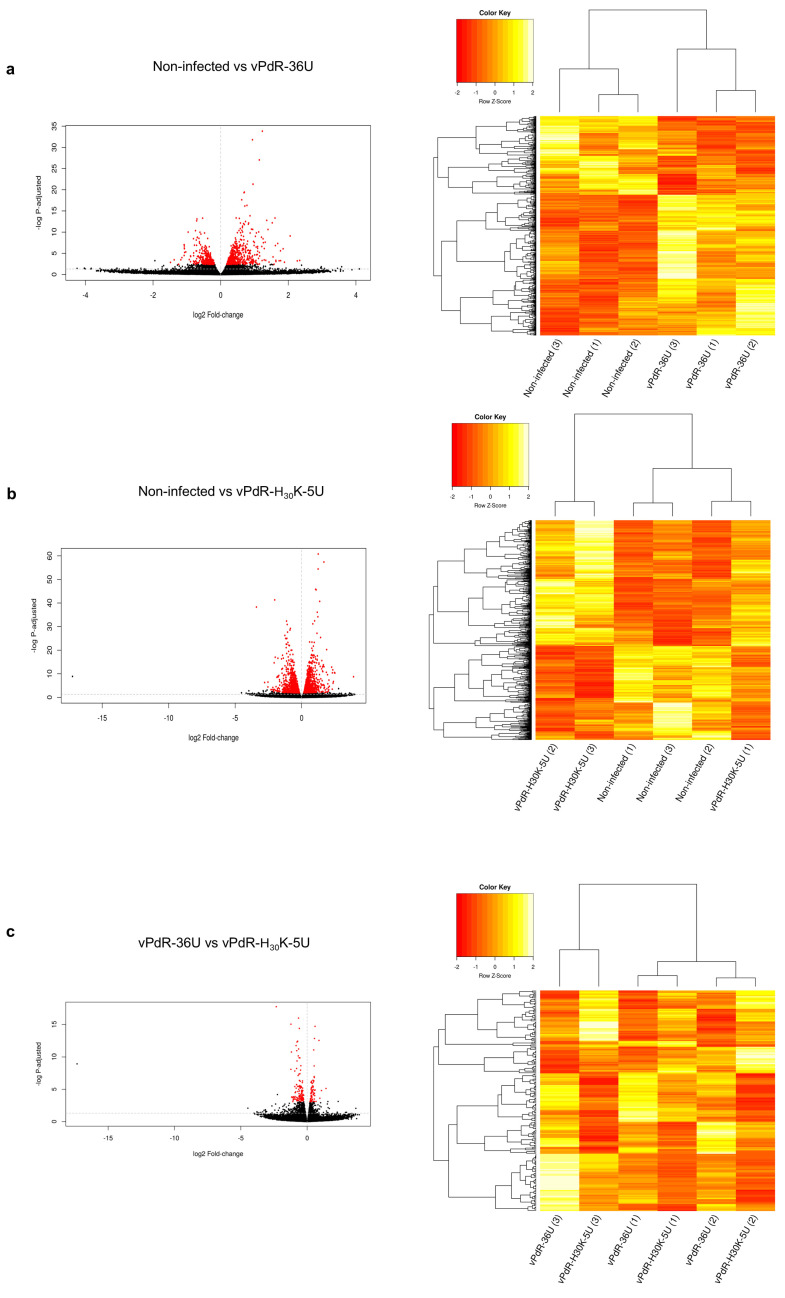
Volcano plot maps and heatmaps of mRNA expression in CSFV-infected BM-APCs. The volcano plot displays the relationship between the fold change and *p* value (represented as −*log p-adjusted*), and the genes differentially expressed with *p*_adj_ < 0.05 are highlighted in red. (**a**) Volcano plot and heatmap for DEGs among vPdR-36U-infected versus noninfected cells. (**b**) Volcano plot and heatmap for DEGs among vPdR-H_30_K-5U-infected versus noninfected cells. (**c**) Volcano plot and heatmap for DEGs among vPdR-36U- versus vPdR-H_30_K-5U-infected cells.

**Figure 4:**
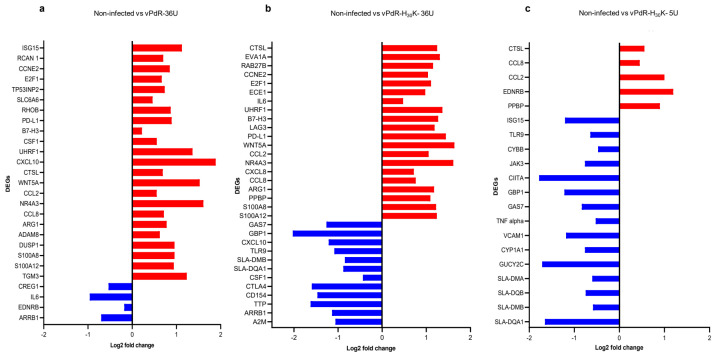
The genes most highly differentially expressed in CSFV-infected BM-APCs. (**a**) The most highly differentially expressed genes in vPdR-36U-infected versus noninfected cells (*p*_adj_ < 0.05). (**b**) The most highly differentially expressed genes in vPdR-H_30_K-5U-infected versus noninfected cells (*p*_adj_ < 0.05). (**c**) The most highly differentially expressed genes in vPdR-36U-infected cells versus vPdR-H_30_K-5U-infected cells (*p*_adj_ < 0.05). The red color shows the overexpressed genes, and the blue color shows the underexpressed genes.

**Figure 5:**
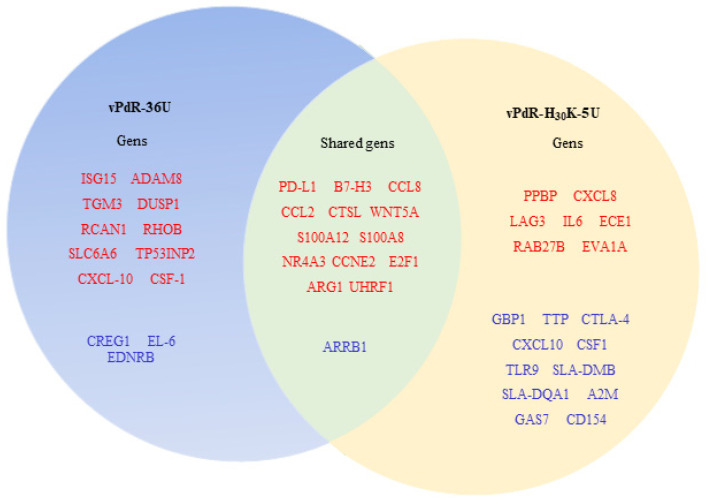
Specific and overlapping DEGs in porcine BM-APCs after 24 h of CSFV infection. The diagram shows the most highly differentially expressed genes found only with vPdR-36U or with vPdR-H_30_K-5U and those found with the two viruses (shared DEGs) in infected BM-APCs. The genes represented in red are the overexpressed genes, and those in blue are the underexpressed genes.

To investigate the differential gene expression between the two groups of CSFV-infected BM-APCs and the PBS-treated cells, the DEG profiles were determined. The read numbers obtained per analyzed samples for each experimental group are shown in the Supplementary Material (Table S2). The volcano plot and heatmap showed the differences found between the experimental groups (Figure 3a–c). The transcriptomic analysis revealed a total of 31,908 expressed genes. When vPdR-36U-infected BM-APCs (Group A) were compared with PBS-treated BM-APCs (Group C), a total of 946 DEGs were detected (Table S3). In this comparison, among the twenty-seven most highly differentially expressed genes, 92% were overexpressed (ADAM8, TGM3, CTSL, DUSP1, RCAN1, RHOB, SLC6A6, TP53INP2, PD-L1, B7-H3, WNT5A, S100A12, S100A8, NR4A3, CCL8, CXCL10, CCL2, ISG15, CCNE2, E2F1, ARG1, UHRF1, and CSF1) and 8% were underexpressed (CREG1, IL6, EDNRB, and ARRB1) (Figure 4a). In addition, among the genes that were specifically modulated after infection with vPdR-36U, 10 were upregulated (ISG15, ADAM8, TGM3, DUSP1, RCAN1, RHOB, SLC6A6, TP53INP2, CXCL-10, and CSF-1) and 3 were downregulated (CREG1, IL-6, and EDNRB) (Figure 4a and Figure 5).

A total of 2643 DEGs were found when the vPdR-H_30_K-5U-infected BM-APCs (Group B) were compared with the PBS-treated BM-APCs (Table S4). Among the most highly differentially expressed genes identified in this comparison, 62.5% were overexpressed (PPBP, CXCL8, CTSL, LAG3, IL6, ECE1, RAB27B, EVA1A, CCNE2, E2F1, UHRF1, B7-H3, PD-L1, WNT5A, CCL2, NR4A3, CCL8, ARG1, S100A12, and S100A8) and 37.5% were underexpressed (GBP1, TTP, CTLA-4, CXCL10, CSF1, TLR9, SLA-DMB, SLA-DQA1, A2M, GAS7, CD154, and ARRB1) (Figure 4b). Similarly, among the genes that were specifically modulated after infection with vPdR-H_30_K-5U, 7 were upregulated (PPBP, CXCL-8, LAG3, IL-6, ECE1, RAB27B, and EVA1A), and 15 were downregulated (GBP1, TTP, CTLA4, CXCL10, CSF1, TLR9, SLA-DMB, SLA-DQA1, A2M, GAS7, and CD154) (Figure 4b and Figure 5).

When comparing the vPdR-36U- and vPdR-H_30_K-5U-infected BM-APCs, a total of 191 DEGs were identified (Table S5). Interestingly, the majority of these DEGs were underexpressed in cells infected with the lethal vPdR-H_30_K-5U virus: SLA-DQA1, GBP1, SLA-DMB, ISG15, GUCY2C, CYP1A1, GAS7, TLR9, CIITA, VCAM1, SLA-DQB, SLA-DMA, JAK3, TNF alpha, and CYBB. Only five genes were overexpressed: PPBP, CCL2, CCL8, EDNRB, and CTSL (Figure 4c). With either of the two CSFV strains, i.e., vPdR-36U that causes chronic CSF or the lethal vPdR-H_30_K-5U, a panel of thirteen overexpressed DEGs and one underexpressed DEG were detected. The shared overexpressed genes were PD-L1, B7-H3, CCL8, CCL2, CTSL, WNT5A, S100A12, S100A8, NR4A3, CCNE2, E2F1, ARG1, and UHRF1. ARRB1 was the only shared underexpressed gene (Figure 5).

**Figure 6:**
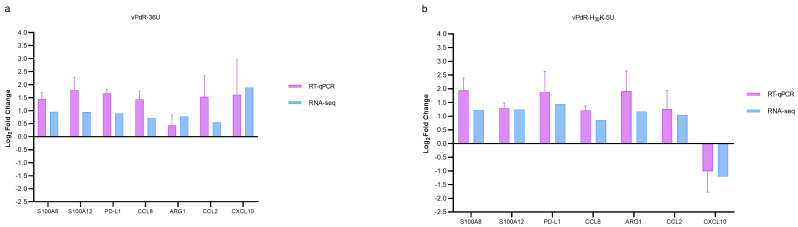
RT-qPCR confirmation of selected gene expression levels. (**a**) RT-qPCR confirmation of the expression levels of seven DEGs in vPdR-36U-infected cells. (**b**) RT-qPCR confirmation of the expression levels of seven DEGs in vPdR-H_30_K-5U-infected cells. The purple bars represent the log2-fold change in the RT-qPCR data, and the blue bars represent the RNA-seq results. The relative expression values expressed as log2-fold changes of the RT-qPCR results were determined by the 2^−ΔΔCt^ method and normalized against the expression levels of the ß-actin gene (see Section 2). The values represent the means of 3 replicate experiments, with the error bars showing the standard deviations.

Furthermore, seven randomly selected DEGs were selected for verification by an RT-qPCR assay to determine the accuracy of the RNA-seq data. In all cases, the RT-qPCR designed for the detection of these genes showed an amplification efficiency of 100%. As shown in Figure 6, the results were consistent between the RT-qPCR and RNA-seq methods. However, the RT-qPCR method tended to overestimate the amount of RNA compared with RNA-seq (Figure 6).

### 3.3. The Capacity of CSFV to Modulate Immune, Antiviral, Cell Cycle, and Metabolic Pathways Depends on the Degree of Viral Virulence

**Figure 7:**
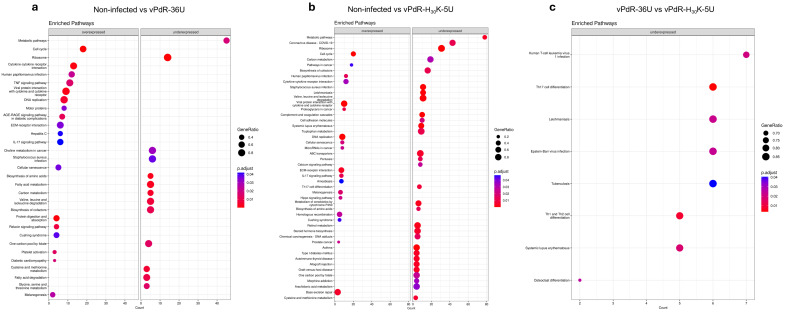
Analysis of enriched KEGG pathways between experimental groups (*p* < 0.05). The most significantly enriched pathways corresponding to sets of activated and suppressed differentially expressed genes are shown. The gene ratio refers to the number of genes observed/total number of genes in that GO term. (**a**) KEGG pathway analysis comparing vPdR-36U-infected versus noninfected cells. (**b**) KEGG pathway analysis comparing vPdR-H_30_K-5U-infected versus noninfected cells. (**c**) KEGG pathway analysis comparing vPdR-36U- versus vPdR-H_30_K-5U-infected BM-APCs. The KEEG database (https://www.kanehisa.jp) was used.

To understand the biological pathways involved after CSFV infection, we mapped the regulated DEGs in the KEGG database (Figure 7a–c). Compared with noninfected BM-APCs, the two viruses used presented similar pathways implicated in the cellular cycle, such as the DNA replication pathway, the cell cycle pathway, and the cellular senescence pathway, which were highly enriched. Moreover, several metabolic pathways were underexpressed in the presence of both vPdR-36U and vPdR-H_30_K-5U: the ribosome pathway, the biosynthesis of amino acid pathway, the carbon metabolism pathway, the valine, leucine, and isoleucine degradation pathway, the biosynthesis of cofactors, and the cysteine and methionine metabolism pathway (Figure 7a,b). Moreover, some antiviral innate immunity pathways, including the cytokine-cytokine receptor interaction pathway, the IL-17 signaling pathway, and the viral protein interaction with cytokine receptor pathway, were more highly expressed in cells infected with either of the two viruses than in noninfected cells (Figure 7a,b). In addition, the extracellular matrix (ECM)–receptor interaction pathway was also enriched in all the CSFV-infected BM-APCs groups compared with the noninfected group (Figure 7a,b and Figure S1).

Unlike the pathways modulated similarly by the two viruses, the nonlethal CSFV vPdR-36U upregulated specifically the TNF signaling pathway, the platelet activation pathway, the hepatitis C pathway, and the protein digestion and absorption pathway. Some of the most relevant genes involved were JAG1, CSF1, TNF, SOCS3, CXCL10, and CCL2. In addition, few underexpressed pathways were observed, all of which are involved in cellular metabolism (Figure 7a). In contrast, in the vPdR-H_30_K-5U-infected cells, most pathways, such as the coronavirus disease pathway, complement and coagulation cascade pathway, ABC transporter pathway, cell adhesion molecule pathway, tryptophan metabolism pathway, type I diabetes mellitus pathway, and systemic lupus erythematosus pathway, were downregulated (Figure 7b). However, four pathways associated with the development of cancer were overexpressed by vPdR-H_30_K-5U (Figure 7b). When the two viruses were compared, the lethal PdR-H_30_K-5U virus dominated in downregulating pathways associated with the immune system, such as the Th17 cell differentiation pathway, Th1 and Th2 cell differentiation pathway, human T-cell leukemia virus 1 infection pathway and Epstein-Barr virus infection pathway (Figure 7c and Figure S1).

## 4. Discussion

CSF continues to be a challenge for the scientific community and for animal health [1]. There are still important gaps in the understanding of CSF pathogenesis related to disease severity. In this study, a transcriptomic approach was used to explore the host pathways that were activated or suppressed, as well as the most highly differentially expressed genes that were involved after porcine BM-APC infection with CSFV strains with different degrees of pathogenicity. To this end, BM-APCs were generated from BMHC precursor cells for eight days by incubation with rpGM-CSF [18]. The transcriptomic profile of the porcine BM-APCs revealed the expression of MerTK, Cadm1, CSF1R, FLT3, and XCR1, which was previously described as a housekeeping gene in these cells [16,25,34]. In addition, according to previous studies, transcripts encoding surface markers (CD14, CD4, CD64) were also detected [25,34]. Despite the different levels of pathogenicity associated with infection with vPdR-36U or vPdR-H_30_K-5U, as well as the different levels of replication of each virus in pigs [4,9], these two viruses replicated at similar levels in BM-APCs. These results agreed with our previous studies in which different replication levels were observed in pigs infected with vPdR-36U or vPdR-H_30_K-5U, although similar replication kinetics were observed in primary porcine BM-APCs and other cell lines [4,9].

In the present study, a panel of overexpressed DEGs was identified after infection with both CSFVs (Figure 5). Within this gene subset, the immune checkpoint inhibitors PD-L1 and B7-H3 were detected. The PD-1/PD-L1 interaction decreases interferon-γ (IFN-γ) and interleukin-2 (IL-2) production under T-cell stimulation in monocyte-derived dendritic cells. PD-1/PD-L1 overexpression correlates with a blockade of the immune response and immune tolerance described in lethal CSF [35]. In addition, B7-H3 upregulation could also facilitate host immune evasion of CSFV by negative immunomodulation, thus promoting CSFV replication and disease severity.

Apoptosis, immunosuppression, and impairment of the host immune response have been described during acute, chronic, and persistent forms of CSF [6,11,36]. In a previous study, a low CD4/CD8 ratio was detected in piglets after postnatal persistent infection with CSFV [15]. These animals also showed nonspecific clinical signs despite permanent viremia and high virus shedding in the absence of IFN-γ- or CSFV-specific antibody responses. In these cases, PD-L1 upregulation could be associated with a decrease in T-cell proliferation and the impairment or blockade of adaptive immunity described in CSF [1,6,11]. Overexpression of Wnt5a was also detected in the presence of both CSFV strains. CSFV causes severe damage to the vascular endothelium and generates a proinflammatory response associated with CSF severity [1]. Wnt5a could be related to this phenomenon, considering its role in the activation of proinflammatory cytokines [37]. Similarly, CCL2, CCL8, NR4A3, ARRB1, ARG1, CTSL, S100A12, and S100A8 were also overexpressed regardless of the virus strain used. Considering their functions, these genes could also contribute to inflammatory response regulation during CSFV infection [38,39]. As mentioned previously, CSFV is involved in apoptosis and immunosuppression [6,11,36], which may be related to the upregulation of the UHRF1, E2F1, and CCNE2 DEGs found during CSFV infection [40,41].

In the case of vPdR-36U, which is related to mild disease (Figure 4 and Figure 5), the upregulation of ISG15 was detected. The IFN-α- and IFN-β-induced ISG15 protein has antiviral activity and is conjugated to intracellular target proteins. Compared with high-virulence strains, CSFV strains associated with mild forms of the disease induce lower IFN-α levels [1,42]. Non exacerbated levels of the type 1 interferon response, such as that generated by low CSFV virulence strains, could mediate the overexpression of ISG15 and promote its antiviral activity, limiting CSFV replication and therefore reducing disease severity in mild forms. The fact that ISG15 was not found among the DEGs associated with the lethal virus vPdR-H_30_K-5U may support the previous hypothesis. The vPdR-36U virus also upregulated CXCL10, which is secreted by several cell types in response to IFN-γ and is involved in chemotaxis, differentiation, and activation of peripheral immune cells [43]. The upregulation of CXCL10 supports the relevant role of IFN-γ in reducing disease severity and CSFV replication observed in mild forms of CSF [11]. ADAM8 plays an important role in modulating inflammation, which could also be involved in controlling the inflammatory response in animals with mild forms of CSF [44]. RCAN1, SLC6A6, RHOB, DUSP, TGM3, and TP53INP2 DEGs, which are related to DNA damage-induced apoptosis, autophagy, the cell cycle, and inflammatory repression, were also upregulated after vPdR-36U infection [45,46].

In the case of the vPdR-H_30_K-5U, the LAG3 gene, encoding an immune checkpoint protein that is also associated with CD8^+^ T-cell exhaustion, was upregulated, supporting its role in the lethal CSF (Figure 5). Interestingly, PPBP, a strong chemoattractant and neutrophil activator, and CXCL8, also known as interleukin-8 (IL-8), a key mediator in the inflammatory response, were overexpressed. These results coincided with an increase in the number of neutrophils (6D10^+^) observed in the BMHC of pigs in acute CSF [6,15]. Interestingly, elevated IL-8 levels were detected in the serum of pigs suffering from lethal CSF after vPdR-H_30_K-5U infection [4]. Additionally, upregulation of the IL-6 gene was detected in vPdR-H_30_K-5U-infected cells, which is consistent with the high IL-6 RNA levels observed after the infection of pigs with a highly virulent CSFV strain [47]. Other genes, such as EVA1A, which is involved in the regulation of autophagy and apoptosis, and ECE1 and Rab27b, which are associated with the growth, invasion, and metastasis of a variety of tumors, were also overexpressed in the context of vPdR-H_30_K-5U infection.

A panel of underexpressed DEGs was identified after vPdRH_30_K-5U infection. Notably, GBP1 has been reported to restrict viral infection, especially against RNA viruses. This gene interacts with the CSFV NS5A protein and suppresses CSFV replication in PK-15-infected cells [48]. Silencing of GBP1 facilitated CSFV replication. Additionally, TLR9, which regulates inflammatory responses, was also downregulated. Likewise, PdR-H_30_K-5U infection also suppressed genes involved in MHC antigen processing and presentation, such as SLA-DQA1 and SLA-DMB. Furthermore, this virus, which proved to be lethal after infection in pigs, also suppressed A2M, whose dysregulation has been linked with tissue inflammation and several cytokine-related diseases [49].

**Figure 8:**
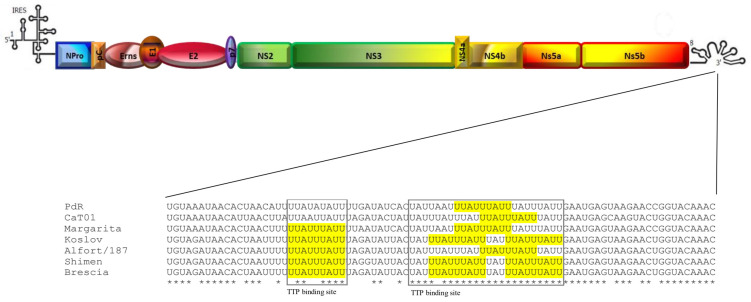
Schematic representation of the CSFV genome with details of the 3′UTR TTP binding sequence. The boxes show the TTP binding site, with the mRNA sequence that interacts with the TTP TZF domain highlighted in yellow.

Moreover, the TTP gene was also downregulated after infection with vPdR-H_30_K-5U. TTP, an RNA-binding protein involved in inflammatory immune responses, binds directly to the 3′UTR of TNF-α mRNA through its CCCH tandem zinc-finger (TZF) domain, resulting in mRNA deadenylation and degradation [50]. Previous studies have shown that TTP-deficient mice have a chronic excess of TNF-α, resulting in severe inflammatory syndrome [51]. Interestingly, the mRNA-binding sequence for the TTP TZF domain (UUAUUUAUU) [52] is conserved in most CSFV strains (Figure 8). Previous reports have shown high expression of TNF-α in sera from infected pigs with high- and moderate-virulence CSFV strains [53]. Although further studies will demonstrate the role of this gene in the inflammatory response to CSFV, these results suggest that TTP may be related to the elevated inflammatory response generated against high-virulence CSFV strains.

CD154, an essential molecule for promoting humoral and cellular immune responses, was found to be downregulated, which may contribute to the impediment of antibody responses observed during the acute form of CSF generated in pigs infected with vPdR-H_30_K-5U [4,9]. Some studies have used CD154 as a vaccine adjuvant to increase the antibody response to CSFV, supporting the role of CD154 in CSF pathogenesis [54]. Interestingly, the expression of CYBB, JAK3, and GUCY2C was also turned off after vPdR-H_30_K-5U infection. CYBB deficiency has been associated with chronic granulomatous disease [55], whereas a lack of JAK3 can generate an increase in proinflammatory cytokines [56]. GUCY2C is involved in the relief of intestinal inflammation and visceral pain during colitis and diarrhea [57], which are symptomatic in the acute and lethal phases of CSF. Thus, the downregulation of these genes may serve as a potential in vitro indicator of virulence and may assist in the definition of endpoint criteria in CSFV animal experiments.

The KEGG pathway enrichment analysis supported the DEG results. Several antiviral pathways related to host immune responses and the regulation of innate immunity, such as the cytokine-cytokine receptor interaction pathway, the IL-17 signaling pathway, and the viral protein-cytokine receptor interaction pathway, were upregulated by the two viruses, which was more prominent for the highly pathogenic vPdR-H_30_K-5U. Previous studies have shown that after CSFV infection, several cytokines and chemokines can induce immune and inflammatory responses and play essential roles in the host antiviral response [1,25]. IL-17 can play a pathogenic role in lung inflammation and acute respiratory distress syndrome associated with viruses such as SARS-CoV-2 and the influenza virus [58] and can promote CD8^+^ T-cell exhaustion [59]. To date, there are no reports on the role of IL-17 in the pathogenicity of CSFV; however, on the basis of these findings, it is conceivable that IL-17 could also be involved in immunosuppression and immune exhaustion during lethal CSF. In this sense, a recent study revealed that the production of IL-17 was significantly increased in the sera of pigs infected with severe African swine fever, another lethal disease affecting swine [60].

Moreover, the Th17 and the Th1 and Th2 cell differentiation pathways were downregulated by vPdR-H_30_K-5U. Both pathways promote CD4^+^ T helper cell differentiation. Ganges et al. [10] reported that the effective induction of MHC class II T-cell response was correlated with protection against CSFV. Related to this, the CD4^+^ T cells decrease drastically during lethal CSFV infection. Thus, downregulation of this pathway by a lethal virus supports the critical role of CD4^+^ T helper cells in the efficient immune response to CSFV [10], which may account for host immune incompetence during the lethal form of the disease. In addition to CD4^+^ T cells, other cell types, such as innate lymphoid cells, natural killer cells, and mast cells, including neutrophils, can also be sources of IL-17 after viral infection [58]. Th17 cells have great plasticity in terms of the production of cytokines in vivo. Although the role of Th17 cells against viral infections is not entirely clear, their reduction has been associated with the progression of diseases in humans [58].

Both CSFVs used in the present study were able to downregulate metabolism-related pathways, although the most dramatic metabolic alterations were found in the cells infected with the highly pathogenic vPdR-H_30_K-5U. A previous study revealed that disruption of these metabolic pathways facilitates the inhibition of energy production and possibly dysbiosis of the intestinal microbiota, which may contribute to the development of CSF [61]. The cell cycle, DNA replication, and cellular senescence pathways were upregulated by both viruses. Cellular stress and chronic inflammation can induce cellular senescence through proinflammatory responses [62]. After vPdR-36U infection, the TNF signaling pathway and platelet activation pathway were upregulated. Interestingly, the TNF signaling pathway is related to the activation and proliferation of different subsets of immune cells, including CD4^+^ T cells. It activates the NF-κB signaling pathway, which plays a critical role in inflammation, immunity, and cell proliferation, among other processes. The increased activity of this pathway also supports the role of T cells against CSFV. The activation of immune responses against vPdR-36U may be correlated with a low viral load and mild clinical disease. The platelet activation pathway is beneficial for hemostasis and for platelet function at sites of vascular wall injury. Endothelial damage caused by CSFV, particularly during chronic and lethal disease stages, has been reported previously [1].

In this study, the human database for functional analysis was used because of the relatively lower quality and completeness of the pig gene ontology databases. This explains why KEGG related to human diseases appeared in the analysis carried out. Given the lack of precision in the annotation of swine-specific databases, the interpretation of some of these pathways needs to be considered cautiously. However, the present study provides relevant information that could be of great value for the future interpretation of KEGG in swine-related diseases. In addition, these results highlight the need to improve the annotation of swine-specific databases to facilitate more precise functional analyses in future studies.

The transcriptional profiles recorded after infection with vPdR-H_30_K-5U were consistent in many parameters with those reported for other lethal viral infections, supporting previously published results on infection with this virus in domestic pigs [4]. Thus, the present study supports the synergistic effect of the 3′UTR and the E^rns^ RNase function in regulating innate immunity against CSFV, favoring virus replication in target tissue and contributing to disease severity [4]. The present study is one of the first to show differential transcriptional profiles after infection of porcine BM-APCs with two related CSFVs, but which generate different pathogenicity in pigs. These findings contribute to a better understanding of CSF pathogenesis and the host factors that determine disease progress and severity. These results could be the basis for further studies that together guarantee new strategies for the development of vaccines, antiviral treatments, and tools for the early detection of immune dysfunction markers against viral infection.

## Data Availability

The original contributions presented in this study are provided within the manuscript and Supplementary Information files. Sequence data have been deposited in the link http://www.ncbi.nlm.nih.gov/bioproject/1047334, available on 1 July 2024.

## Supplementary Materials

The following supporting information can be downloaded at: https://www.mdpi.com/article/10.3390/v17020160/s1, Figure S1. Enrichment map based on the ten most enriched KEGG pathways. (a) Network of interacting genes of the most enriched KEGG pathways comparing vPdR- 36U-infected versus noninfected BM-APCs. (b) Network of interacting genes of the most enriched KEGG pathways comparing vPdR-H_30_K-5U-infected versus noninfected BM-APCs. (c) Network of interacting genes of the most enriched KEGG pathways comparing vPdR-36U-infected BM-APCs versus vPdR-H_30_K-5U-infected BM-APCs. Edges are sized based on the number of genes shared by the connected pathways. Node size represents the number of genes in the pathway. Table S1. RT-qPCR primers used for the validation of differential expressed genes. Table S2. The number of readings per sample analyzed for each experimental group. Table S3. The differentially expressed genes in vPdR-36U-infected versus non-infected cells. Table S4. The differentially expressed genes in vPdR-H30K-5U-infected versus non-infected cells. Table S5. The differentially expressed genes in vPdR-36U-infected versus vPdR-H30K-5U-infected cells.
