# Supplementary material for: Gene Expression Signatures of Porcine Bone Marrow-Derived Antigen-Presenting Cells Infected with Classical Swine Fever Virus
Source: Viruses. 2025 Jan 24;17(2):160. doi: 10.3390/v17020160 (PMC11860178; doi:10.3390/v17020160)
Supplement: Supplementary file 1 [file viruses-17-00160-s001.zip › Table S1.pdf]

Supplementary Table S1. RT-qPCR primers used for the validation of differential expressed genes.

Gene	Foward sequence	Reverse sequence	Probe	GenBank access number
β-actina	5' ATCATGAAGTGCGACGTCGA 3'	5' TGATCTTCATCGTGCTGGG 3'	6FAM - TCTACGCCAACAACACGGTGC - TAMRA	AH006582.2
S100A8	5' TACCACGCCATCTACGCG 3'	5' ACGCCCACCTTGATCACC 3'	6FAM - TGTTAGAGACGGAGTGTCC - TAMRA	NM_001160271.3
S100A12	5' TGCATTGAAGGGTGAACGC 3'	5' TTCTTGAGGGTGTTGGGC 3'	6FAM - AGCTGGAAGACCATCTGG - TAMRA	NM_001160272.1
PD-L1	5' AAATTGCAAGATGCAGGG 3'	5' TAAGTTCATGTTCAGAGG 3'	CY5- GCTTGATCAGCTATGGTGG - NONE	NM_001025221.1
CCL8	5' TGGTCAATGGAAAGATCC 3'	5' TTCATGGAATTCTGGACC 3'	6FAM - AGCTACACAAGAATCACC - TAMRA	FJ_156752.1
ARG1	5' AGCGGTGAGAAATATTGG 3'	5' TTTCTCAATGCTGTAGGG 3'	6FAM - TCAAGTCACAATCCATCG - TAMRA	NM_214048.2
CCL2	5' TGATCTTCAAGACCATCG 3'	5' TTAGGGCAAGTTAGAAGG 3'	6FAM - TCCATAAGCCACCTGG - TAMRA	NM_214214.1
CXCL10	5' TCTGAGTGGAACTCAAGG 3'	5' TGATCTCAACATGTGGGC 3'	6FAM - AGAACTGTTCGCTGTACC - TAMRA	NM_001008691.1
