# Supplementary figures and images for: Gene Expression Signatures of Porcine Bone Marrow-Derived Antigen-Presenting Cells Infected with Classical Swine Fever Virus
Source: Viruses. 2025 Jan 24;17(2):160. doi: 10.3390/v17020160 (PMC11860178; doi:10.3390/v17020160)

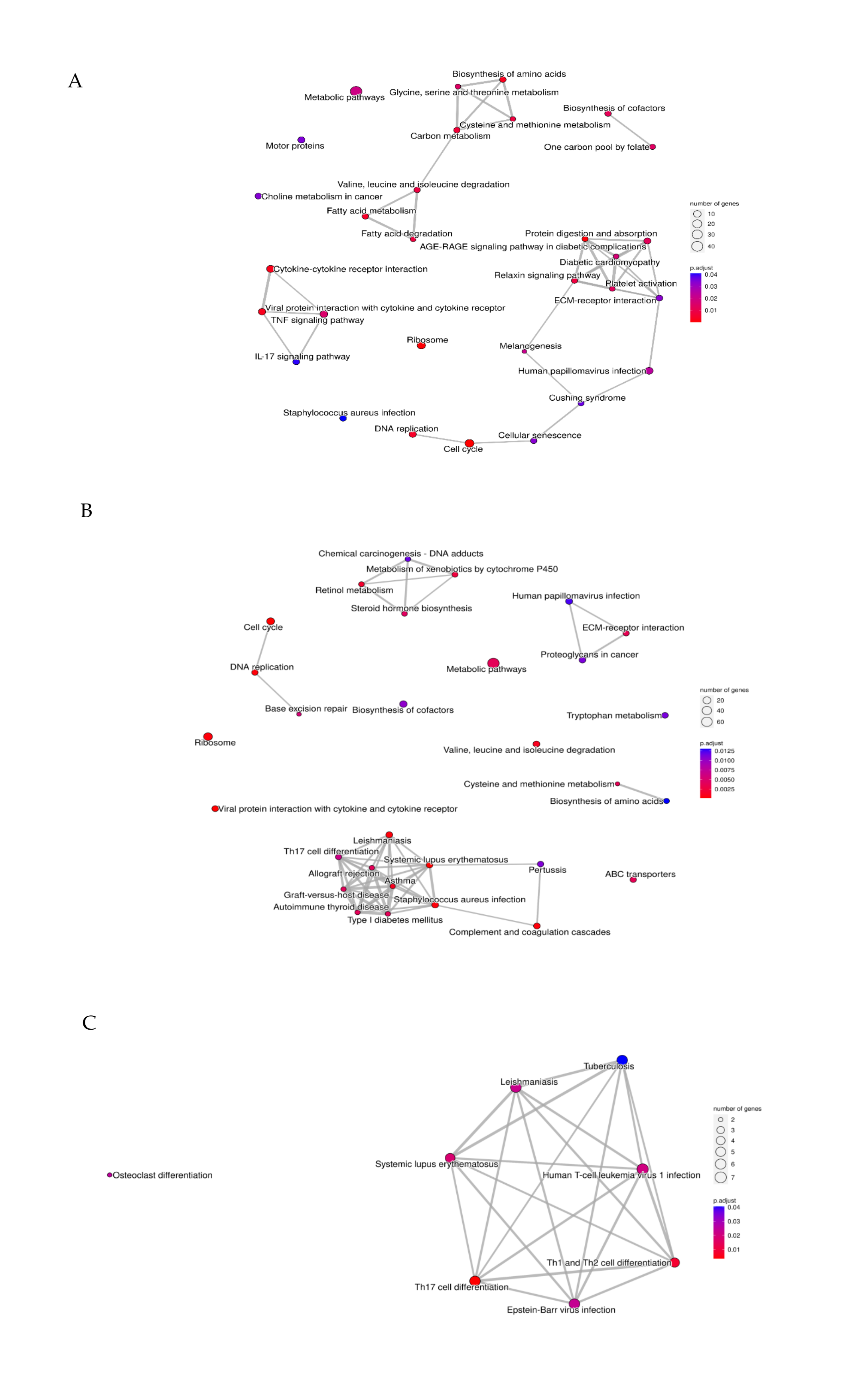

Supplement: Supplementary file 1 [file viruses-17-00160-s001.zip › Figure S1.tiff]
